# Influence of high glucose on mesangial cell-derived exosome composition, secretion and cell communication

**DOI:** 10.1038/s41598-019-42746-1

**Published:** 2019-04-18

**Authors:** Antônio da Silva Novaes, Fernanda Teixeira Borges, Edgar Maquigussa, Vanessa Araújo Varela, Marcos Vinicios Salles Dias, Mirian Aparecida Boim

**Affiliations:** 1 Renal Division, Department of Medicine, Federal University of São Paulo, São Paulo, Brazil; 2 International Research Center, AC Camargo Cancer Center, São Paulo, Brazil

**Keywords:** Mechanisms of disease, Molecular biology, Diabetic nephropathy

## Abstract

Mesangial cells stimulated with high glucose (HG) exhibit increased intracellular angiotensin II (AngII) synthesis that is correlated with the upregulation of AngII target genes, such as profibrotic cytokines. The intracrine effects of AngII can be mediated by several molecules transferred to other cells via exosomes (Exos), which play a key role in cellular communication under many physiological and pathological conditions. The aim of this study was to investigate the effects of exosomes derived from HG-stimulated human mesangial cells (HG-HMCs) on normal unstimulated HMCs. Exosomes from HMCs (C-Exos) and HG-HMCs (HG-Exos) were obtained from cell culture supernatants. HMCs were incubated with C-Exos or HG-Exos. HG stimulus induced a change in the amount but not the size of Exos. Both C-Exos and HG-Exos contained angiotensinogen and renin, but no angiotensin converting enzyme was detected. Compared with HMCs treated with C-Exos, HMCs treated with HG-Exos presented higher levels of fibronectin, angiotensinogen, renin, AT_1_ and AT_2_ receptors, indicating that HG-Exos modified the function of normal HMCs. These results suggest that the intercellular communication through Exos may have pathophysiological implications in the diabetic kidney.

## Introduction

Intercellular communication plays a key role in regulating physiological and pathophysiological processes. Exosomes, which are released by most cells, constitute an important pathway in cell communication^1,2^. Exosomes can be taken up and deliver many signaling molecules to neighboring or distant cells^3–8^.

The molecular content of exosomes is determined by secretory cells and is regulated by the cellular physiology; however, it can be altered under pathophysiological conditions^9^. Thus, exosomes can be produced and secreted with altered contents in response to many stimuli, such as changes in oxygen tension, modifications in the glucose supply and cytotoxic drugs^7,10^.

The involvement of exosomes in cell communication has been demonstrated in many pathologies, such as tumor growth and metastasis formation^11–13^, as well as other pathologies, including hepatic^6^, neurodegenerative^14^ and renal^6^ diseases.

Diabetes mellitus and its secondary manifestations are directly involved in the onset of renal damage, representing one of the main causes of chronic kidney disease^15–17^. Mesangial cell activation in response to hyperglycemia results in the increased synthesis of the mesangial matrix and the mesangial expansion contributing to progressive glomerular damage. In addition, *in vitro* studies using mesangial cells (MC) in culture showed that excess glucose is a potent stimulus for intracellular synthesis and the release of AngII^18,19^, contributing to the intrarenal AngII overproduction, a hallmark diabetic nephropathy (DN)^20^. AngII plays a pivotal local role in the progression of renal disease by its proinflammatory and profibrotic effects^20–24^.

Chronic kidney disease has a progressive nature, and cellular communication plays a crucial role in this process^20,25–28^; however, the participation of exosomes in this process has not been fully explored. We hypothesized that high-glucose activated MCs could influence neighboring healthy cells through cell-to-cell communication via exosomes. Thus, the present study evaluated the biology and contents of the exosomes secreted by high glucose-activated human MCs (HMCs) and examined their potential role in the transfer of information to control cells. Exosomal cargo was analyzed in terms of fibrotic molecules and the components of the renin angiotensin system (RAS). The contents of the exosomes released by HMCs stimulated by high glucose were also evaluated and whether these exosomes would be able to alter the function of unstimulated target cells was determined. The obtained results are consistent with the hypothesis that high glucose-activated HMCs could influence neighboring healthy cells through cell-to-cell communication via exosomes.

## Results

### Characterization of exosomes from HMCs cultured under control and high-glucose conditions

**Figure 1:**
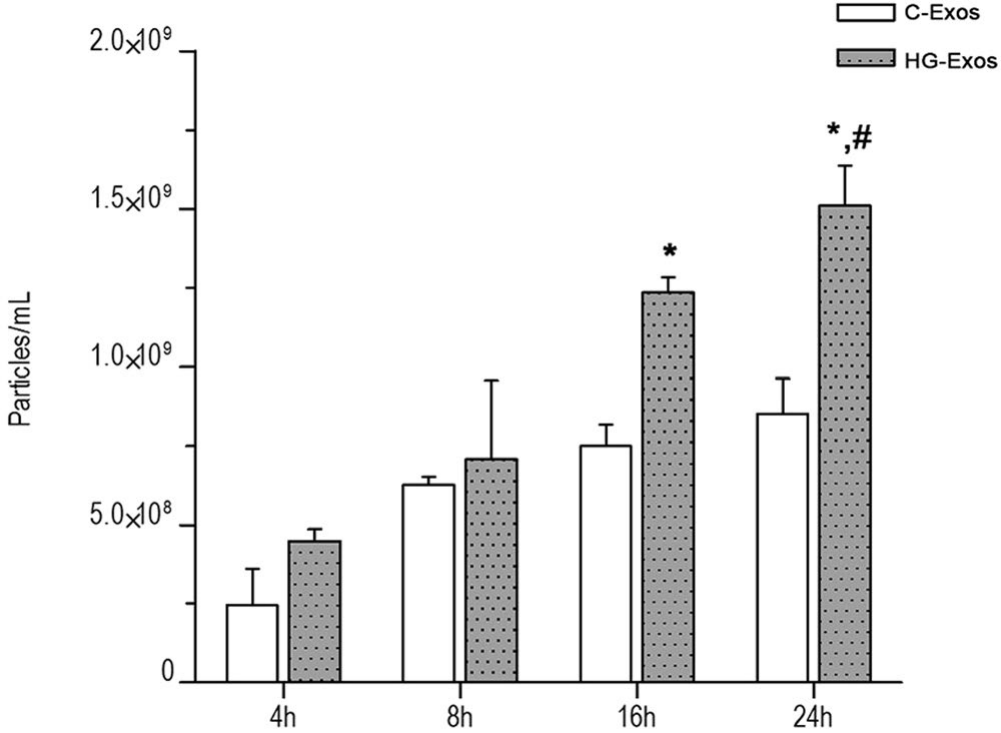
HG-treated HMCs secreted more exosomes than normal HMCs. Quantification of exosomes was normalized to the cell number by using the Countess^®^ Automated Cell Counter. The number of exosomes was quantified by using Nanoparticle Tracking Analysis. Six separate flasks were used, and the exosome number was quantified from three video recordings. Error bars are plotted as s.e.m. *p < 0.05 versus the HG-Exos 4 hr group, ^#^*p* < 0.05 versus C-Exos. C-Exos group: exosomes derived from normal glucose-treated HMCs (Student’s *t*-test); HG-Exos group: exosomes derived from HG-treated HMCs.

Initially, we verified whether HMCs could release exosomes and examined the role of high-glucose in this process. Exosomes released by HMCs were collected and evaluated at 4, 8, 16 and 24 hr. The results showed that HG-stimulated HMCs released a higher number of exosomes compared to unstimulated cells with significance after incubation for 16 and 24 hr (Fig. 1).

**Figure 2:**
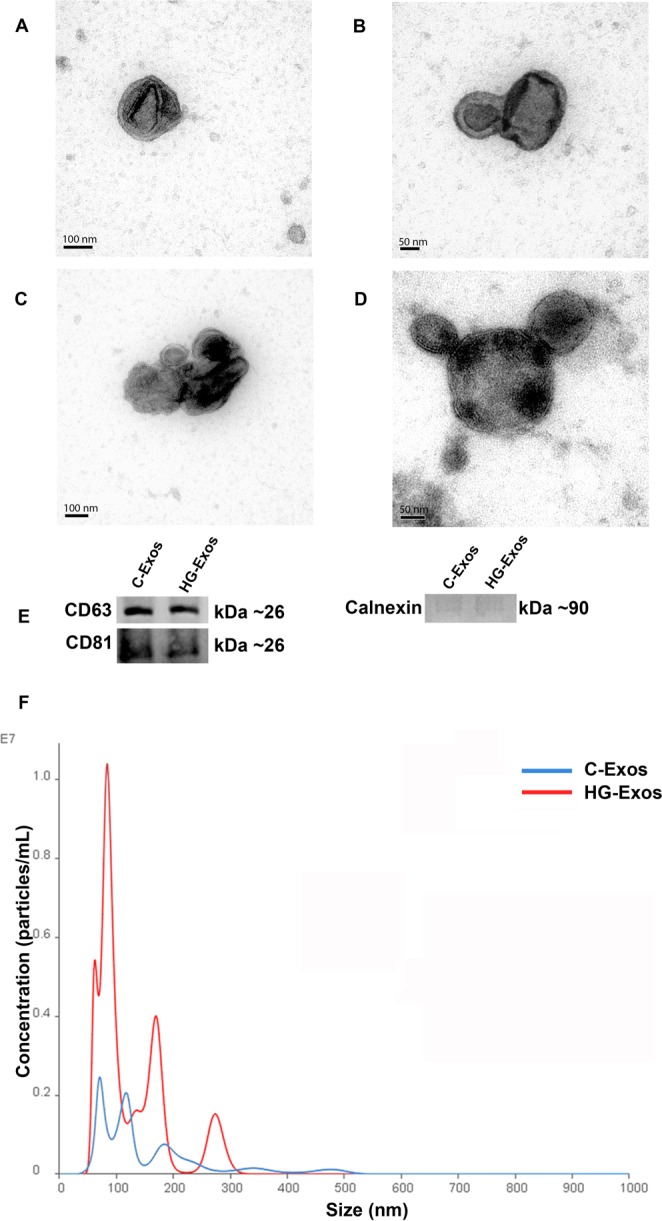
Exosomes characterization. (**A**) C-Exos. (**B**) HG-Exos. (**C**) C-Exos and (**D**) HG-Exos clusters with variable sizes. Images were obtained by transmission electron microscopy. Cup-shaped structures of 30–150 nm in size were identified as exosomes. Magnification: ×150,000. (**E**) Western blot analysis for exosome markers CD63 and CD81. (*n* = 3 for each index). (**F**) Histogram representing the profile of nanoparticle size by Nanoparticle Tracking Analysis. The values represent the means ± SEM, and all values are representative of at least three independent experiments.

Exosomes were characterized according to their shapes and sizes by transmission electron microscopy (TEM). As shown in Fig. 2A, the spherical vesicles presented typical cup-shaped exosomes (Fig. 2B). There were no morphological differences between the C-Exos and HG-Exos groups. Figures 2C,D show vesicles of variable sizes. The presence of the exosomal markers CD63 and CD81 tetraspanins was evidenced by western blotting (Fig. 2E). Finally, the particle size was analyzed by screening nanoparticles, and as shown in Fig. 2F, both groups showed similar profiles for particle size, with a mean of 135 nm, compatible with exosome size. However, a higher number of particles were identified in the HG-Exos group compared to the C-Exos group, as also shown in Fig. 1.

These results indicated that HMC produced and released extracellular vesicles of sizes and shapes compatible with exosomes. Cellular stimulation with a high glucose concentration did not alter the shapes and sizes of the exosomes but significantly influenced the number of particles released.

### Exosomes were internalized by HMCs

**Figure 3:**
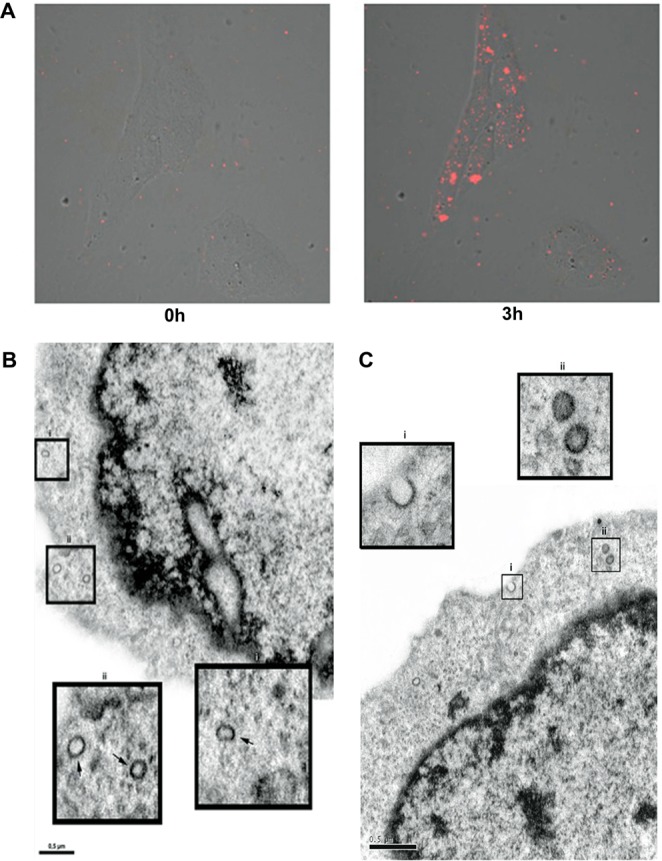
High glucose-stimulated HMC-derived exosomes can be internalized by normal HMCs. Exosomes isolated from the culture supernatants of HG treated-HMCs were labeled with fluorescent dye PHK26 and incubated with normal HMCs for 3 h. (**A**) Fluorescence intensity image immediately and at 3 hr after the addition of PKH26-labeled GF-Exos (red). (**B**) Transmission electron microscopy illustrates C-Exos incorporated by a normal HMC (arrow). Bi and Bii: Expansion of endocytosed exosomes (arrow). (**C**) Transmission electron microscopy showed HG-Exos incorporated by normal HMC. C_I_: HG-Exos being incorporated by a healthy HMC. C_II_: Expansion of the endocytosis of HG-Exos.

To evaluate the ability of normal HMCs to take up exosomes, we labeled HG-Exos with PKH26 (red fluorescent lipidic marker). The cellular uptake of HG-Exos was observed under confocal laser microscopy (Fig. 3A). After the 3-hr incubation period, PKH26-labeled HG-Exos were localized in the cytoplasm of HMCs, indicating that exosomes were internalized by the cells.

Uptake of C-Exos (Fig. 3B) and HG-Exos (Fig. 3C) by control HMCs was also verified by electron microscopy. Vesicle incorporation into cell can be observed (black arrows), shown in detail with a larger size. The fluorescence intensity of the images of HG-Exos labeled with PKH26 (red) at different depths (z) is demonstrated in Supplemental Fig. 3A. The addition of the dye alone did not produce intracellular labeling, which indicates that intracellular labeling observed in the Fig. 3A is due to internalization of exosomes into cell. (Supplemental Fig. 3B).

### Content of Exosomes

**Figure 4:**
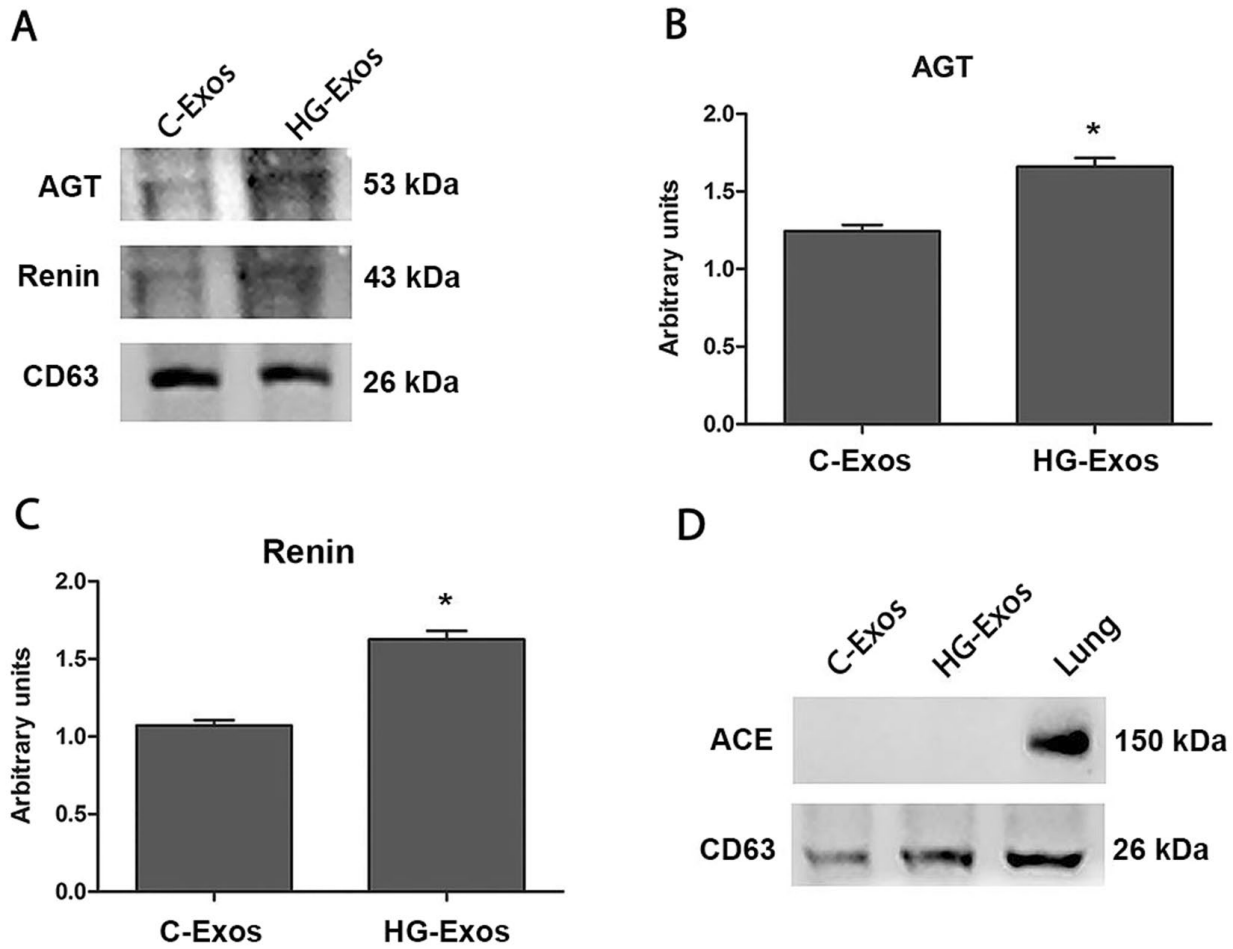
RAS components present in the exosomes. Protein extracts were obtained from C-Exos and HG-Exos. The image show in A is representative of three independent experiments. Exosomal protein expression for angiotensinogen (**B**), renin (**C**) and ACE (**D**) was determined by western blotting. ACE was not detected in the exosomal extract protein (**D**). The results are expressed as the means ± standard error. p ≤ 0.05: * vs C-Exos.

Since mesangial cells are an important intrarenal source of the components of the renin angiotensin system (RAS), the presence angiotensinogen (AGT), renin and angiotensin-converting enzyme (ACE) in the C-Exos and HG-Exos was evaluated by western blotting. The results showed that the C-Exos protein extract constitutively contained renin and angiotensinogen proteins (Fig. 4A), but no ACE was detected, as shown in Fig. 4D. The positive control for ACE expression was obtained in mouse lung extract. Densitometric analysis of the bands (Fig. 4B,C) showed that renin and angiotensinogen proteins were increased in HG-Exos compared to C-Exos.

**Figure 5:**
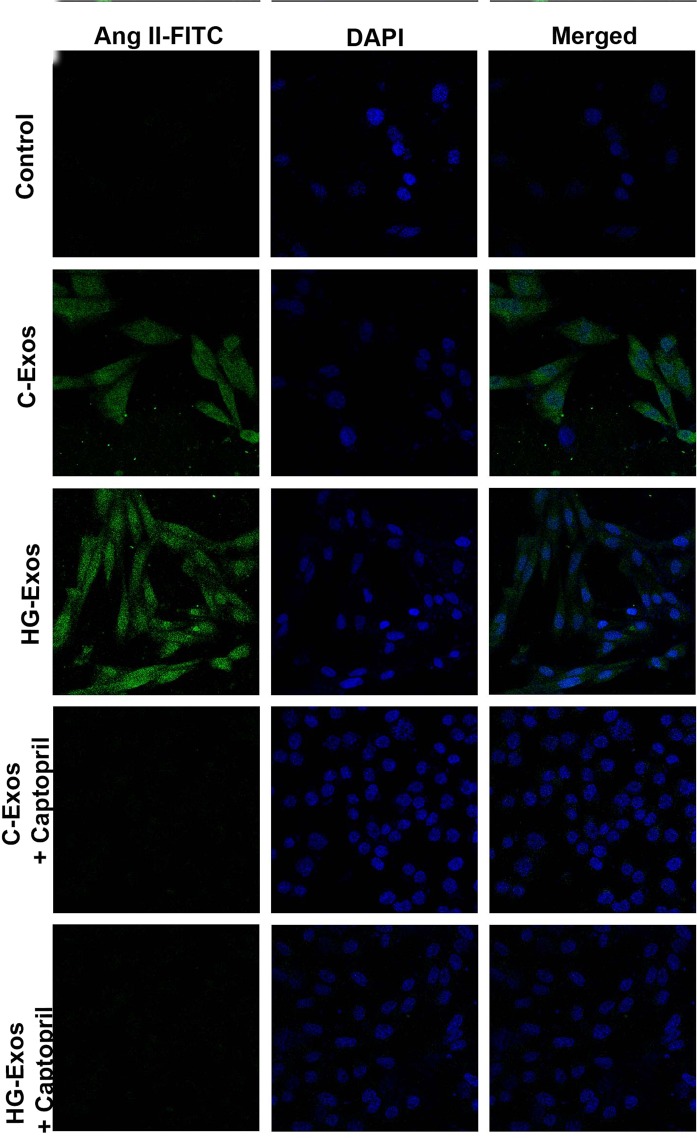
Synthesis of AngII induced by C-Exos and HG-Exos in ACE-CHO. Chinese Hamster Ovary cells transfected with angiotensin converting-enzyme (ACE-CHO) were incubated with C-Exos or HG-Exos. After 24 hr, AngII staining was evaluated by immunofluorescence under confocal microscopy. For each 1 million ACE-CHO cells, 2 × 10^8^ particles/mL of C-Exos or HG-Exos was added in the presence and absence of captopril (ACE inhibitor). The staining for AngII (green) was not observed in ACE-CHO cells that were not treated with exosomes (control). The staining for AngII (green) was observed in ACE-CHO exposed to either C-Exos or HG-Exos. AngII staining was not observed in the presence of captopril. The images are representative of three independent experiments. Magnification of 630x. The nuclei were stained with DAPI (blue).

To examine the ability of HMC-derived exosomes to induce the *de novo* synthesis of intracellular AngII, we used CHO-K1 cells since this cell line does not express RAS components^29^. Considering that exosomes derived from HMCs did not contain ACE, another group of CHO cells was transfected with ACE (ACE-CHO). CHO-K1 and ACE-CHO cells were incubated with C-Exos or HG-Exos in the presence or absence of the ACE inhibitor captopril. As expected, ACE-CHO expressed ACE (Fig. 5A). After incubation for 24 hr, the presence of AngII in ACE-CHO was analyzed by immunofluorescence under a confocal laser microscope. CHO-K1 and ACE-CHO cells alone did not express AngII (Fig. 5B, upper panel). In contrast, ACE-CHO cells exposed to either C-Exos or HG-Exos showed AngII staining, indicating that AngII was synthesized from the contents released by the exosomes (Fig. 5B, middle panels). AngII labeling was not observed in the presence of captopril (Fig. 5B, lower panels). The same experiment was performed with CHO-K1 (non-transfected cells). As expected, the presence of ACE was not observed in these cells (Supplemental Fig. 4A). In addition, no AngII staining was observed in CHO-K1 treated with either C-Exos or HG-Exos (Supplemental 4B). These results indicate that C-Exos and HG-Exos can contribute to AngII synthesis in target cells.

### HG and HG-Exos increased the expression of AT_1_ and AT_2_ receptors

**Figure 6:**
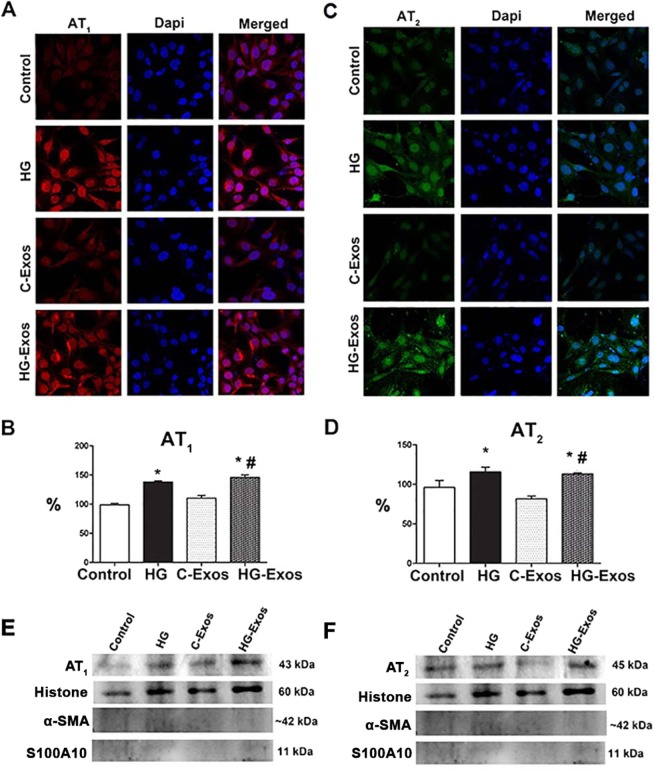
Effect of high glucose, C-Exos or HG-Exos on the expression of AT_1_ and AT_2_ receptors in intact HMC and in isolated nuclei. HMCs were incubated with HG (30 mM), C-Exos or HG-Exos. After 24 hr, the intact cells were labeled with antibodies anti-AT_1_ (**A**) or anti-AT_2_ (**C**), and the staining was detected by immunofluorescence under confocal microscopy. The protein expression for AT_1_ (**B**) and AT_2_ (**D**) in the nuclear extract was determined by western blot. The image is representative of three independent experiments. Representative bands of nuclear AT_1_ (E) and AT_2_ (F) expression. Expression of the nuclear protein histone and absence of α-smooth muscle actin (α-SMA) and cytoplasm membrane S100A10 proteins confirmed the purity of the samples. The results were expressed as the mean ± standard error of the mean (SEM). P < 0.05: * vs Control; # vs C-Exos.

To assess whether HG-Exos would induce HMC to increase the expression of AT_1_ and AT_2_ receptors in both whole HMC and in the isolated nuclei, we incubated HMCs with HG, C-Exos or HG-Exos for 24 hr. The presence of the receptors in whole cells was assessed by immunofluorescence under confocal microscopy (Fig. 6A,B), while the presence of receptors in isolated nuclei was assessed by western blotting (Fig. 6C,D). Faint labeling for both AT_1_ (Fig. 6A) and AT_2_ (Fig. 6B) was observed; however, in the presence of either HG or HG-Exos, the labeling for both receptors was stronger. The same profile was observed in nuclei isolated from HMCs exposed to both HG or HG-Exos (Fig. 6C,D). Figure 6E showed representative bands of AT_1_ and AT_2_. Expression of the nuclear protein histone and absence of α-smooth muscle actin (α-SMA) and cytoplasm membrane S100A10 proteins confirmed the purity of the samples.

### Activation of HMC by HG-Exos

To determine whether exosomes are involved in the cellular communication contributing to the perpetuation of the mesangial cell activation in the diabetic milieu, we added exosomes derived from HG-stimulated HMCs to the culture medium of healthy HMCs. The production of RAS components (AGT, renin and AT_1_ and AT_2_ receptors), cell proliferation and mesangial matrix production were estimated at 24 hr after the exposure of HMC to C-Exos or HG-Exos.

**Figure 7:**
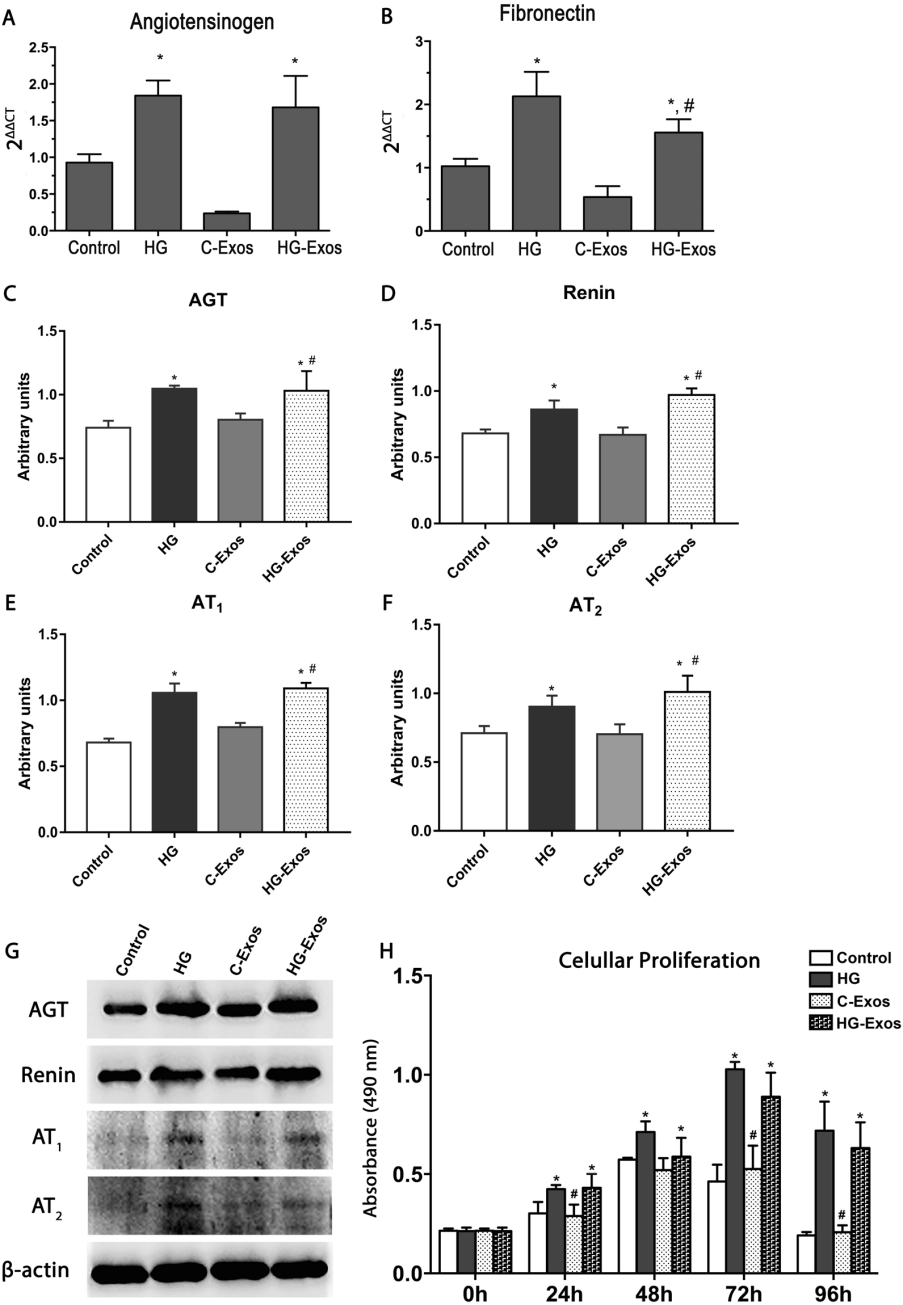
Activation of normal HMC by high glucose and HG-Exos. Normal HMCs were incubated with high glucose, C-Exos or HG-Exos. After 24 h, the gene expression for angiotensinogen (AGT) (**A**) and fibronectin (**B**) was evaluated. Protein expression of AGT (**C**), renin (**D**), AT_1_ (**E**) and AT_2_ (**F**) in the cell extract was determined by western blotting. The image is representative of three independent experiments and is shown in the lower panel, while the densitometric analysis is shown in the graph. (**G**) Representative bands of AGT, Renin, AT_1_ and AT_2_ expression. β-actin was used as a loading control. (**H**) HMCs were stimulated with high glucose or treated with C-Exos or HG-Exos. After 0, 24, 48, 72 or 96 h, the cell proliferation was evaluated by 3-(4,5-Dimethylthiazol-2-yl)-2,5-Diphenyltetrazolium Bromide (MTT) (n = 3). The results are expressed as the means ± standard error of the mean (SEM). p < 0.05: * vs Control **p < 0.01 vs control group. ***p < 0.001 vs control group.; # vs C-Exos.

The gene expression of AGT (Fig. 7A) and fibronectin (Fig. 7B) was significantly increased in both HG-stimulated HMCs and HMCs exposed to HG-Exos. As osmotic control, 30 mM mannitol was added in HMC instead of glucose for 24 h (Mannitol group). From culture medium of HMC stimulated with mannitol, the exosomes (Mannitol-exos) were collected and added in normal HMCs and the gene expression of AGT and fibronectin was evaluated. Mannitol-exposed cells also presented an increase in AGT and fibronectin, but the mean value obtained did not differed significantly from control cells (Supplemental Fig. 5). AGT (7C), renin (7D), AT_1_ (7E) and AT_2_ (7F) proteins presented the same profile. Figure 7G showed representative bands of AGT, renin, AT_1_ and AT_2_. C-Exos treatment did not induce changes in either mRNA or protein expression. Cell proliferation was increased in HMCs incubated with HG or with HG-Exos in all periods at 0, 24, 48 and 72 hr (Fig. 7H).

## Discussion

In diabetes, whether exosomes can mediate the communication among the mesangial cells and participate in the pathogenesis of DN remains unclear. To further define the role of HMC-derived exosomes, we used an *in vitro* model to test the hypothesis that exosomal signaling is responsive to the increased extracellular glucose concentration in HMCs. The main findings of the present study were as follows: (1) HMCs likely use intercellular communication mechanisms involving extracellular vesicles, including exosomes; (2) this mechanism is preserved in HMCs maintained under *in vitro* conditions; (3) exosomes purified from HMCs stimulated with high glucose presented higher amounts of RAS components; and 4) HG-Exos transmit the source cell information to unstimulated control cells that in turn respond with alterations similar to those induced by HG.

HMCs constitutively secreted exosomes, and the high-glucose stimulus induced an increase in the number of exosomes. A similar effect was also observed in trophoblastic cells^7^, suggesting that high levels of glucose can interfere with exosome bioactivity. This phenomenon may contribute to the signal amplification induced by the high-glucose milieu, where cells that react to HG stimulus can activate non-responsive cells, perpetuating the injury signaling.

The results showed that Exos secreted by HMCs cultured under standard conditions constitutively contained the components of the RAS, except ACE. Although certain cell surface proteins are found in the membrane of exosomes, we did not find the ACE in the exosomes. ACE is a typical extracellular membrane enzyme, thereby explaining the absence of ACE since exosomes are structures whose biosynthesis occurs from the intracellular compartment^30,31^, unlike the microvesicles released from the plasma membrane. Moreover, this result is in consistent with the mean size of the observed particles, which was compatible with exosomes^32^. These results suggest that information on intracellular RAS activity may be exchanged among cells through exosomes signaling, even under physiological conditions.

However, many stimuli, such as exposure to alcohol^33^, thermal shock^34^, oxidative stress^35^, hypoxia^4,36^, and acidic pH^37^, can modify the Exos contents, and in the present study, HMCs exposed to high glucose concentrations secreted Exos enriched with higher levels of renin and AGT than the Exos contents released by control HMCs. This finding is of particular importance since the increased intrarenal activity of RAS has been implicated in the pathogenesis of diabetic nephropathy^38,39^. Mesangial cells are a potential source of RAS components in the kidney and HG stimulates the intracellular synthesis of AngII in these cells^18,19^. Moreover, an increase in the local levels AngII has been implicated in the early manifestations of diabetic nephropathy characterized by mesangial expansion and the production of the profibrotic molecules such as fibronectin. Here, we demonstrated that the RAS activation induced by glucose in HMCs can be transmitted to other cells by exosomes. In fact, normal non-stimulated HMCs exposed to HG-Exos presented the same response observed in whole HMCs exposed to HG. This information transmission via exosomes was confirmed in non-RAS component-expressing CHO cells transfected with ACE. ACE-CHO cells displayed AngII labeling only when exposed to the Exos from HMCs, indicating that AngII was produced from the content released by the exosomes. Although Exos did not contain ACE, HMCs express ACE mRNA^18^ and exhibit ACE activity^40^, enabling HMCs to increase AngII synthesis from exosomal AGT and renin.

Hyperglycemia has a cytotoxic effect on mesangial cells^41–44^. In this study, we demonstrated that Exos derived from HMCs stimulated by the HG concentration influenced the phenotype of non-stimulated HMCs. Control HMCs stimulated by the HG-Exos showed increased AGT and renin contents, indicating that the cargo of HG-Exos was efficiently delivered to control cells, and contributed to increase RAS components in unstimulated target cells. As a phenotype response, it was observed a overproduction of fibronectin in HMC exposed to HG-Exos, an cell proliferation increase in target cells, and a similar effect was observed in whole HMCs stimulated with HG^45,46^.

We previously observed^47^ that the AngII receptors AT_1_ and AT_2_ are present in the cytoplasm and the nuclear membrane of HMCs, and these intracellular receptors may be involved in some of the intracellular responses of AngII, including gene activation. These responses are defined as the intracrine actions of AngII^48–50^. In the present study, we observed that HG induced the overexpression of both AT_1_ and AT_2_ receptors in the nuclear membranes of HMCs, and interestingly, the exosomes derived from HG-stimulated HMCs induced the same effect in the target cells. The pathophysiological relevance of the intracrine effect of AngII is not completely understood, but we speculated that the biological effects observed in HMCs stimulated with HG-Exos were triggered, at least in part, by the intracellular action of AngII.

Although the results obtained in this study strongly suggest that the information on RAS activity can be transmitted from cell to cell by exosome signaling in HMCs, these results do not exclude the transmission of other proteins, mRNAs or microRNAs present in the Exos from HMCs stimulated by the HG concentration, which could also play an important role in intercellular communication and cell phenotype modification. Taken together, these results indicate that the Exos from cells stimulated by high glucose change the phenotype of normal cells, suggesting that this mechanism of cellular communication may be important in the transmission and spread of changes to cells and tissues that are not directly affected.

In summary, the main results obtained in the present study suggest that (1) HMCs under standard culture conditions release Exos; (2) glucose is a factor that can regulate the release and interfere with the contents of Exos secreted by HMCs; and (3) Exos isolated from HMCs stimulated by glucose induced the production of profibrotic and biologically active substances with normal HMCs.

Exosome communication is active in HMCs and may have implications in the pathophysiology of diabetic nephropathy.

## Experimental Procedures

### Mesangial cell culture

Immortalized human mesangial cells (HMC) were provided by Dr. Bernhard Banas (Nephrology Center, University of Munich, Germany)^51^. The cells were grown at 37 °C in plastic flasks in Dulbecco’s Modified Eagle’s medium (DMEM, Invitrogen Corporation, Gaithersburg, MD, USA) containing 10% fetal bovine serum (FBS), penicillin (50 U/ml) and 2.6 g HEPES. The culture flasks were maintained in a 95% air and 5% CO_2_ humidified environment. At confluence, HMCs were exposed to culture medium containing no FBS for 24 hr according to the following experimental groups: the control group, cultured in DMEM containing a standard concentration of 5 mM D-glucose, and the High Glucose (HG) group, cultured for 48 hr in DMEM containing 30 mM D-glucose. Subsequently, the culture media were collected, and the exosomes were isolated from both groups. Then, HMCs were exposed to exosomes from control cells (Control-Exos group) or from HG-stimulated cells (HG-Exos group). After incubation for 24 h, the culture media were aspirated, and the cells were analyzed.

### Exosome extraction

Differential centrifugation was used to extract exosomes from the serum-free HMC culture supernatants as previously described^8^. In brief, HMC culture supernatants were collected and sequentially centrifuged at 300 × *g* for 10 min, 2,000 × *g* for 20 min, and 10,000 × *g* for 30 min to remove lifted cells, cellular debris and large vesicles. The cleared samples were then subjected to ultracentrifugation at 100,000 × *g* for 120 min twice at 4 °C to pellet the exosomes (Exos). The resulting exos were resuspended in a small amount of PBS for direct use in subsequent studies.

Exos were obtained from supernatant after 2 hr of ultracentrifugation. As previously published methodologies diverge in the time of ultracentrifugation, a preliminary experiment was carried out to standardize the time to 1 or 2 hr of ultracentrifugation. As shown in Supplemental Fig. 1A, compared with ultracentrifugation for 1 hr, ultracentrifugation for 2 hr resulted in a larger number of particles. The size of the Exos was not altered by the ultracentrifugation time (Supplemental Fig. 1B). These findings confirm the presence of HMCs-derived Exos in our preparations and indicate that HG stimulated a higher release of Exos by HMCs.

### Quantification of exosome particles

The size and concentration of exosomes from HMCs stimulated or not with high glucose was determined by nanoparticle tracking analysis (NTA) to measure the rate of Brownian motion to particle size by using a Malvern NanoSight (NS300) system (Worcestershire, UK) that tracks individual particles and the Stokes-Einstein equation to calculate their diameters. Three replicates of diluted aliquots of vesicle fractions (1 mL in PBS) were injected into the machine’s specimen chamber, and the vesicles were tracked and measured for 30 sec, three times for each sample, at a constant flow rate.

### Transmission electron microscopy

#### Cells

Cultured HMCs were fixed in 4% formaldehyde (PFA; Polysciences Inc., 18814) and 2% glutaraldehyde (Sigma-Aldrich, 340855) in 0.1 M phosphate buffer, pH 7.4. After fixation, the samples were rinsed several times with PBS, followed by post-fixation in 1% osmium tetroxide (EMS, 19150) in phosphate buffer for 1 h. The samples were rinsed with PBS for 15 min and dehydrated through a series of graded ethanol washes ranging from 70% to 100%. The samples were subsequently immersed in an propylene oxide: epon mixture (1:1) and polymerized in pure epon (Polysciences, Inc. 02334-500) at 60 °C for 48 h. Ultrathin sections were stained with uranyl acetate and lead citrate and imaged at 80 kV on a JEOL 1200 EX II transmission electron microscope (JEOL, USA). Images were acquired with a GATAN 781 camera (USA).

#### Exosomes

Frozen, isolated exosomes were resuspended in 1% buffered formaldehyde, adsorbed onto formvar-carbon-coated electron microscopy grids (EMS, FCF200H-Cu), and fixed with a mixture of 2% formaldehyde for 20 min. A 50-µL drop of PBS was placed on a sheet of parafilm, and grids were transferred with the sample side facing down on the drop for 2 min. The grids were transferred to a 50-µL drop of 1% glutaraldehyde for 5 min prior to transfer to a 100-µL drop of distilled water for 2 min. Negative staining was performed by placing the grid over a 50-µL drop of uranyl-oxalate solution for 5 min (4% uranyl acetate, EMS, 22400-4; 0.15 M oxalic acid, Sigma-Aldrich, 75688 pH 7) before transfer to a 50-µL drop of methyl-cellulose-UA (4% uranyl acetate and 2% methyl cellulose for 10 min, Sigma-Aldrich, M6385, in a ratio of 100 µL:900 µL) on a glass dish covered with parafilm on ice. The grids were removed, and excess fluid was blotted gently onto Whatman no.1 filter paper. The grids were dried, stored in appropriate grid storage boxes, and subsequently observed under a JEOL 1200 EX II transmission electron microscope at 80 kV. Images were acquired with a GATAN 781 camera (USA).

#### Incorporation of exosomes

First, HMCs stimulated with high glucose-derived exosomes were labeled with red lipophilic fluorescent dye PKH26 (Sigma-Aldrich, St. Louis, MO) according to the manufacturer’s instructions. After labeling, the exosomes were incubated with normal cells for approximately 3 hr at 37 °C and viewed by a confocal microscope.

#### Western blot analysis

Total protein was purified from the cells and isolated nuclei extracts. The cell and nuclei suspensions were homogenized in ice-cold lysis buffer containing 50 mM Tris (pH 8.0), 150 mM NaCl, 1% Nonidet P-40, 0.5% sodium deoxycholate, 0.1% sodium dodecyl sulfate (SDS), 2.5 mM ethylenediaminetetraacetic acid (EDTA), 1 mM phenylmethylsulfonyl fluoride (PMSF) and 44 mM o-phenanthroline (all reagents from Sigma-Aldrich Chemical Co., USA). The protein concentration was determined by the Lowry method (DC Protein Assay; Bio-Rad Laboratories Inc., Richmond, CA, USA). Equal amounts of total extracted proteins (50 μg) were separated by SDS-polyacrylamide gel electrophoresis (SDS-PAGE) and then transferred onto a nitrocellulose membrane (Amersham Pharmacia Biotech, Piscataway, NJ, USA). Blots of nuclear protein were subjected to immunoblot analyses with primary polyclonal antibodies against AT_1_ (1:1000;) and AT_2_ (1:1000) receptors (Immuny, Brazil), the nuclear protein histone (1:1000; Sigma, USA), the cytosolic protein α-smooth muscle actin (α-SMA) (1:1000; Sigma-Aldrich, USA) and the plasma membrane protein S100A-10 (1:1000; Sigma, USA) to verify the purity of the sample.

Blots of cell protein were subjected to immunoblot analyses with the primary polyclonal antibodies against Angiotensinogen (1:1000; Sigma-Aldrich, USA), Renin (1:1000; Sigma-Aldrich, USA), AT_1_ and AT_2_.

Exosomal protein expression was verified by antibodies against CD63 (1:100, Abcam), CD81 (1:1000 Santa Cruz), renin, angiotensinogen and ECA (1:1000; Sigma-Aldrich, USA).

Immunodetection was achieved by incubating the blots in horseradish peroxidase-conjugated (HRP) anti-rabbit or anti-mouse secondary antibody (1:30,000 dilution). The protein bands were visualized using the Immobilon Western HRP substrate (Millipore). The obtained bands were quantified using the Luminescent Image Analyzer-LAS 4000 and Image Gauge V3.1 software (Fuji Photo Film Co, Tokyo, Japan).

#### Expression of AT_1_ and AT_2_ in nuclei of HMC

HMCs express AT_1_ and AT_2_ receptors in the nuclear membrane^52^, which mediate the nuclear effects of AngII. Thus, we investigated whether HG-HMC-derived exosomes could modify the distribution and number of nuclear receptors. Intact nuclei were isolated according to the protocol of Ho YF *et al*.^53^. In brief, after confluence, HMCs were trypsinized, transferred to a tube and centrifuged at 1200 *g* for 5 min. The cell pellet was resuspended into 4 mL of 0.25 M sucrose/TKM buffer (50 mM Tris-HCl, 5 mM MgCI, 25 mM KCl, pH 7.5). The suspension was centrifuged again at 600 *g* for 5 min. The suspension was divided into aliquots and transferred to separate ultracentrifuge tubes containing 2 volumes of 2.3 M sucrose/TKM. The contents were mixed by inversion. The mixture was carefully underlaid with 4 mL of 2.3 M sucrose/TKM and overlaid with TKM to fill the tube. The tubes were centrifuged at 25,000 *g* for 10 min. The pellet at the bottom of the tube contained the nuclei. The nuclear pellet was washed by resuspension in TKM, followed by centrifugation at 900 *g* for 5 min at 4 °C, and the resulting pellet contained highly purified nuclei, which were resuspended in phosphate-buffered saline (PBS) buffer and stored at −80 °C for future use. For protein analyses, the nuclear protein extract was purified using the Nuclei Isolation kit according to the manufacturer’s instructions (Sigma-Aldrich, St. Louis, MO, USA). The nuclear protein extract was used in western blotting.

The expression of AT_1_ and AT_2_ receptors was assessed by immunofluorescence in whole cells and isolated nuclei from HMCs by western blotting under the following conditions: I: Control: cells treated with standard medium; II: cells treated with high-glucose medium; III: cells exposed to C-Exos; and IV: cells exposed to HG-Exos.

HMCs were grown on LabTec^®^ coverslips in a standard culture medium and fixed in 3.5% formaldehyde for 15 min at room temperature. The cells were rinsed with PBS and subsequently permeabilized with 0.2% Triton X-100 for 10 min. Whole HMCs cells were incubated overnight with primary antibodies against AT_1_ (1:100), AT_2_ (1:100) receptors (Immuny, Brazil). The primary antibody was washed out, and the cells were incubated with secondary antibody (rabbit anti-rabbit diluted 1:50 in PBS) conjugated to tetramethylrhodamine isothiocyanate (TRITC) or fluorescein isothiocyanate (FITC) for 40 minutes at room temperature (in a dark chamber) and then rinsed with PBS. Finally, the cells were incubated with DAPI (4′,6-diamidino-2-phenylindole dihydrochloride, Invitrogen Life Technologies Inc., Gaithersburg, MD, USA) diluted 1:50 in PBS for 15 min to label the nucleus. The reagents were washed out, and the coverslips and slides were mounted with pH 9.0 glycerin/500 mM Na_2_CO_3_ and 500 mM NaHCO_3_ (MERK SA Chemical Industries, Rio de Janeiro, RJ, Brazil). The images were obtained using a confocal microscope (Zeiss LSM 510 META, Carl Zeiss Inc., Jena, Germany) and were digitalized using ZEN software (Zeiss, Germany).

#### mRNA expression by RT-PCR

The mRNA expression levels of fibronectin, angiotensinogen and β-actin were estimated by quantitative RT-PCR. The total RNA was purified from HMCs by using a commercial phenol and guanidine isothiocyanate-cesium chloride method (TRIzol, Gibco BRL, Rockland, MD, USA) according to the manufacturer’s instructions. A volume of 2 μg of total RNA was treated with DNase (RQ1 RNase-free DNase; Promega, Madison, WI, USA) to avoid genomic DNA contamination. The RNA pellet was resuspended in RNase-free water and reverse transcribed into cDNA by the addition of a mixture containing 0.5 mg/mL oligodeoxythymidylate (oligo-d(T)), 10 mM dithiothreitol (DTT), 0.5 mM deoxynucleoside triphosphates (Amersham Pharmacia Biotech, Uppsala, Sweden) and 200 U reverse transcriptase enzyme (SuperScript RT; Gibco BRL). RT-PCR amplification was performed using the GeneAmp 5700 Sequence Detection System (Applied Biosystems, Foster City, CA, USA), with specific primers for each molecule as follows (forward and reverse, respectively): fibronectin (5′ acccaattccttgctggtatca 3′ and 5′gtatattcggttcccggttcca 3′); angiotensinogen (5′ tcaaaccaggagaggaac 3′ and 5′ agatggcgaacaggaaggg 3′); and β-actin (5′ cctctatgccaacacagtgc 3′ and 5′ acatctgctggaaggtggac 3′). The amplified products were monitored using the SYBR Green I intercalating dye (Molecular Probes, Eugene, OR, USA), which exhibits high fluorescence upon the binding of double-stranded DNA. The fluorescence for each cycle was quantitatively analyzed using the Sequence Detection system (Applied Biosystems). At the end of each PCR run, a melting curve was produced by increasing the temperature from 60 °C to 95 °C at a rate of 2 °C/min, and the fluorescence was measured every 15 s. This technique provided verification of the presence of a single amplification product. The relative gene expression was calculated using the previous PCR conditions under which the amplification curve was logarithmic. The mRNA expression levels were normalized to β-actin, and the results were expressed as arbitrary units, with the control cells as the reference sample.

#### Cell proliferation using MTT assay

HMCs were plated onto 96-well plates at a density of 10^3^ cells/well. After attachment for 24 hr, the culture medium was replaced according to each group, and the cells were cultured for an additional 24, 48, 72, or 96 hr. The supernatants were discarded, and 200 μL of culture medium containing 0.5% 3-(4,5-dimethylthiazol-2-yl)-2,5-diphenyltetrazolium bromide (MTT) was added to each well. After a 4-hr incubation period, the culture medium was carefully removed, and 100 μL of dimethyl sulfoxide (DMSO; Sigma-Aldrich) was added to each well. The cells were placed in an incubator and vortexed at a low speed for 2 min to fully dissolve the formazan crystals. The optical density (OD) of each well was measured at 490 nm using the EON microplate reader (BIOTEK, Winooski, Vermont, USA).

#### CHO-K1 and CHO-ACE culture

Wild-type Chinese Hamster Ovary cells (CHO-K1) were used as a negative control for AngII synthesis, as these cells do not express any RAS components^29^, including AT_1_ and AT_2_ receptors. CHO-K1 cells were provided by Dr. Guacyara da Motta (Bioquemistry Departament, Federal University of São Paulo, Brazil). This cell line was cultured in Ham’s F-12 nutrient mixture medium (Invitrogen Corporation, Gaithersburg, MD, USA).

CHO-K1 cells were transfected with the angiotensin-converting enzyme (ACE) gene and were kindly provided by Dr. Dulce Casarini (Department of Medicine, Federal University of São Paulo). ACE-transfected CHO-K1 cells (ACE-CHOs) were cultured at 37 °C in culture plates with DMEM, containing 10% FBS, penicillin (50 U/ML) and 2.6 g HEPES. Both cell lines were cultured in plastic plates in a humidified incubator containing 5% CO_2_ at 37 °C. Culture media were supplemented with 10% (v/v) fetal bovine serum (FBS) and penicillin (50 U/mL).

### Statistical analysis

The results are expressed as the means ± standard error of mean (SEM). Statistical significance was determined through one-way analysis of variance (ANOVA), followed by either Newman–Keuls test or Tukey’s test. P-values less than 0.05 were considered statistically significant. Statistical analysis and graph construction were performed using GraphPad Prism software version 5.00 for Windows (GraphPad Software, San Diego, CA, USA).

## Supplementary information

Supplemental Figures
