# Author Correction: Influence of high glucose on mesangial cell-derived exosome composition, secretion and cell communication

**DOI:** 10.1038/s41598-020-58753-6

**Published:** 2020-01-29

**Authors:** Antônio da Silva Novaes, Fernanda Teixeira Borges, Edgar Maquigussa, Vanessa Araújo Varela, Marcos Vinicios Salles Dias, Mirian Aparecida Boim

**Affiliations:** 1 Renal Division, Department of Medicine, Federal University of São Paulo, São Paulo, Brazil; 2 International Research Center, AC Camargo Cancer Center, São Paulo, Brazil

Correction to: *Scientific Reports* 10.1038/s41598-019-42746-1, published online 18 April 2019

This Article contains an error in Figure 3C, where the incorrect image was used for the transmission electron microscopy image of high-glucose exosomes incorporated by normal human mesangial cells. The correct Figure 3 appears below as Fig. 1.

**Figure 1:**
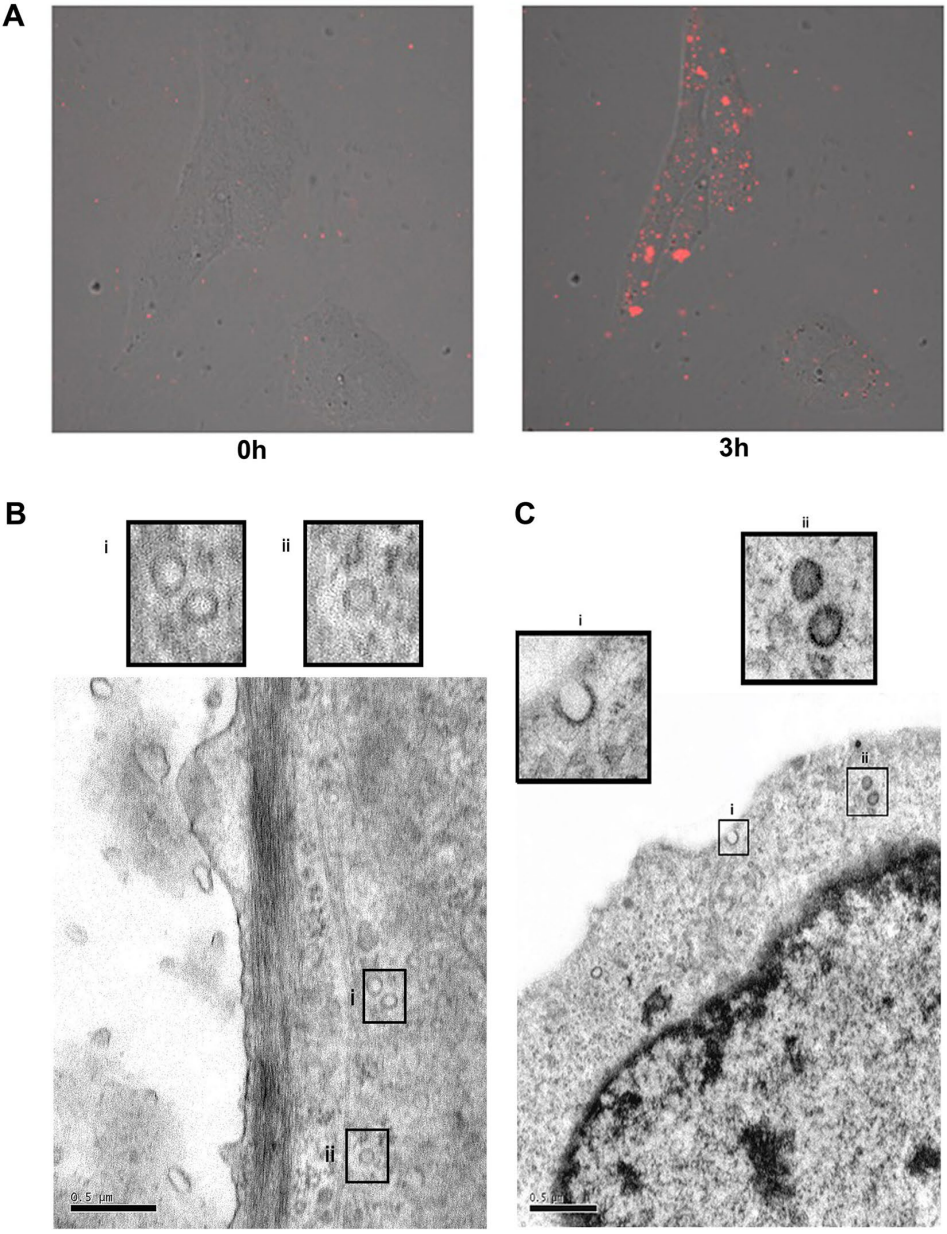
.

