# Supplementary material for: Influence of high glucose on mesangial cell-derived exosome composition, secretion and cell communication
Source: Sci Rep. 2019 Apr 18;9:6270. doi: 10.1038/s41598-019-42746-1 (PMC6472340; doi:10.1038/s41598-019-42746-1)

Supplemental Figures

Influence of high glucose on mesangial cell-derived exosome composition, secretion and cell communication

[1]Antônio da Silva Novaes, [1]Fernanda Teixeira Borges, [1]Edgar Maquigussa, [1]Vanessa Araújo Varela, [2]Marcos Vinicios Salles Dias, [1]Mirian Aparecida Boim

[1]Renal Division, Department of Medicine, Federal University of São Paulo,
[2]International Research Center; AC Camargo Cancer Center.

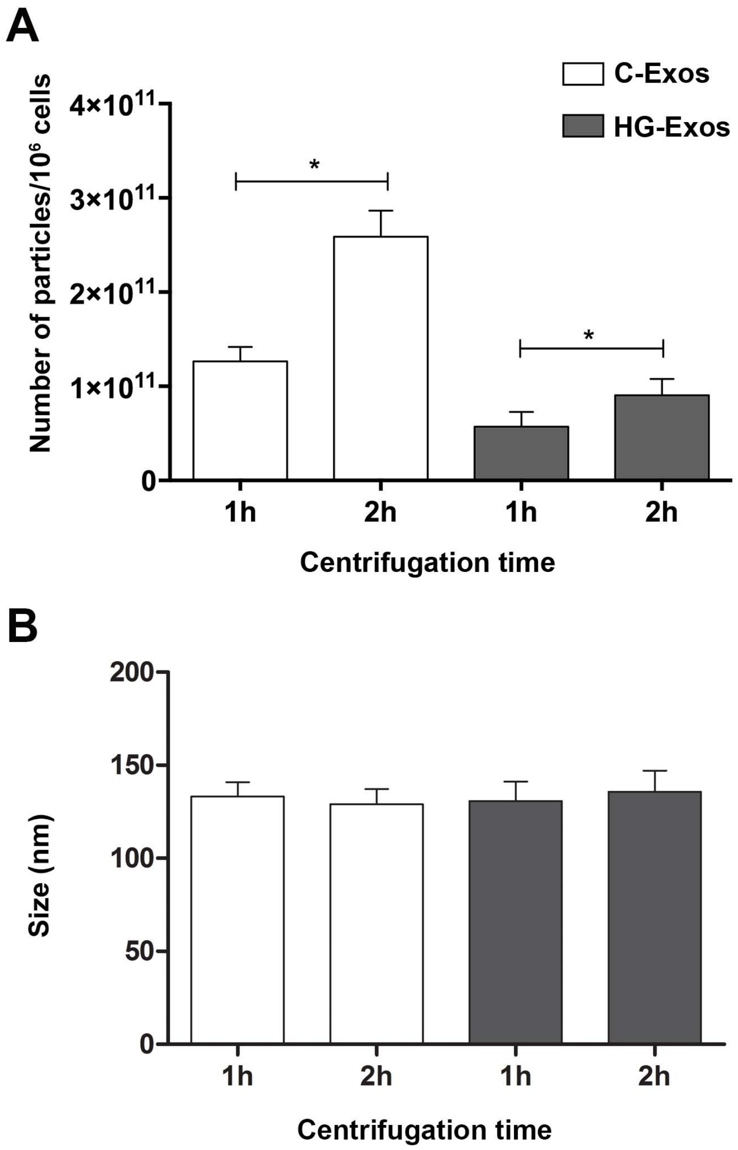

Supplemental Figure 1 – Number of particles obtained after ultracentrifugation for 1 or 2 hours. Data are shown as mean ± standard error (SE). *$p < 0.05$.

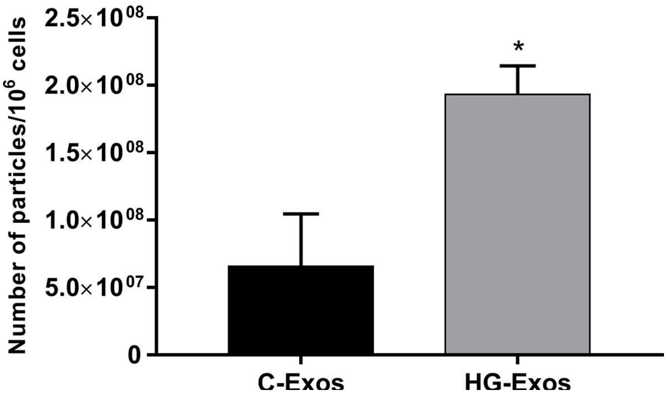

Supplemental Figure 2 – Concentration of exosomes released by 10^6 cells. HMCs from different passages were grown on 6-well plates in culture medium supplemented with 5 or 30 mM glucose. After 24 hr of incubation, the exosomes were isolated from the culture medium with 5 mM (c-Exos) or 30 mM (HG-Exos) and the adhered HMCs were trypsinized and counted in an automatic counter. Six independent experiments were performed. Data are shown as mean ± standard error (SE). $*p < 0.001$.

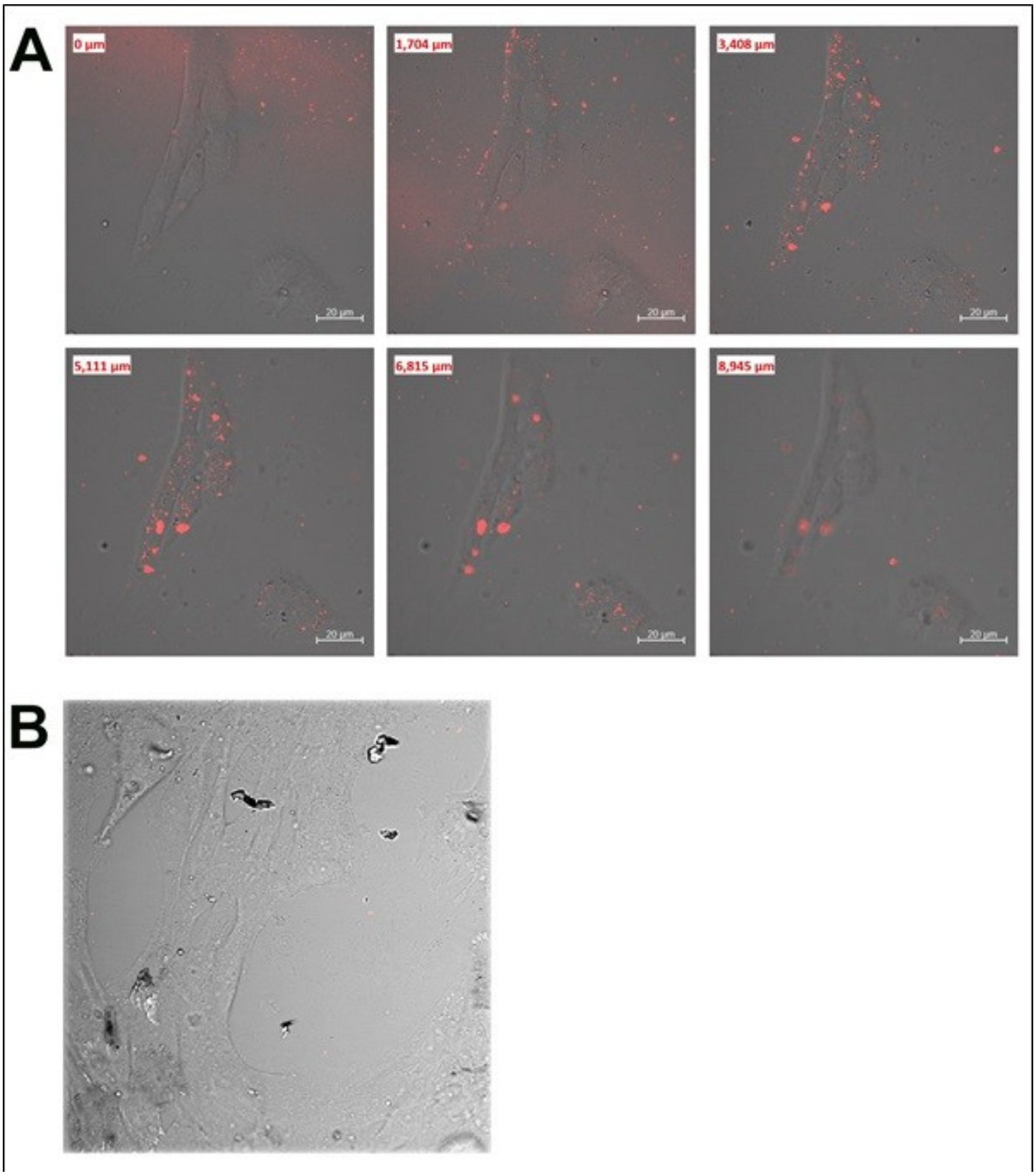

Supplemental Figure 3 – Incorporation of HG-Exos by normal HMC. A - Fluorescence intensity images after 3 hr of addition and HG-Exos labeled with PKH26 (red) at different depths (z) of normal HMC. B – Negative control. The addition of 50 uM of PKH26 labeling was not observed in cells after 3hr of incubation.

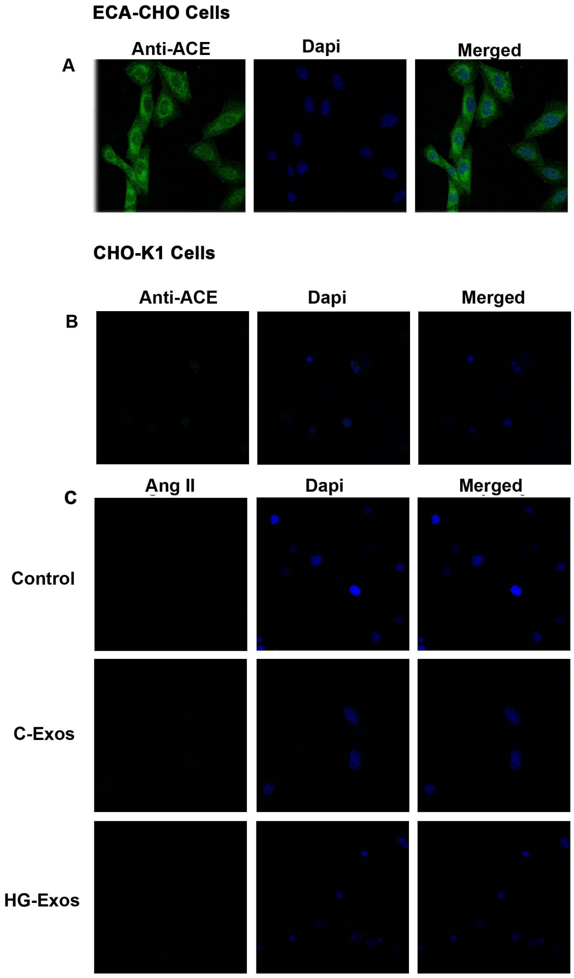

Supplemental Figure 4 – Synthesis of AngII in CHO-K1 incubated with C-Exos and HG-Exos. Chinese hamster ovary cells were incubated with C-Exos and HG-Exos. After 24 h, we evaluated AngII synthesis by immunofluorescence under confocal microscopy. A – Staining for ACE (green) was observed in ACE-CHO cells. B – Staining for angiotensin converting enzyme (ECA) (green) was not observed in CHO-K1 cells. C – AngII labeling was not observed in untreated CHO-K1 (control). The labeling for AngII (green) was not observed in CHO-K1 treated with C-Exos and HG-Exos. The images are representative of three independent experiments. Magnification of 630x. The nuclei were stained with DAPI (blue).

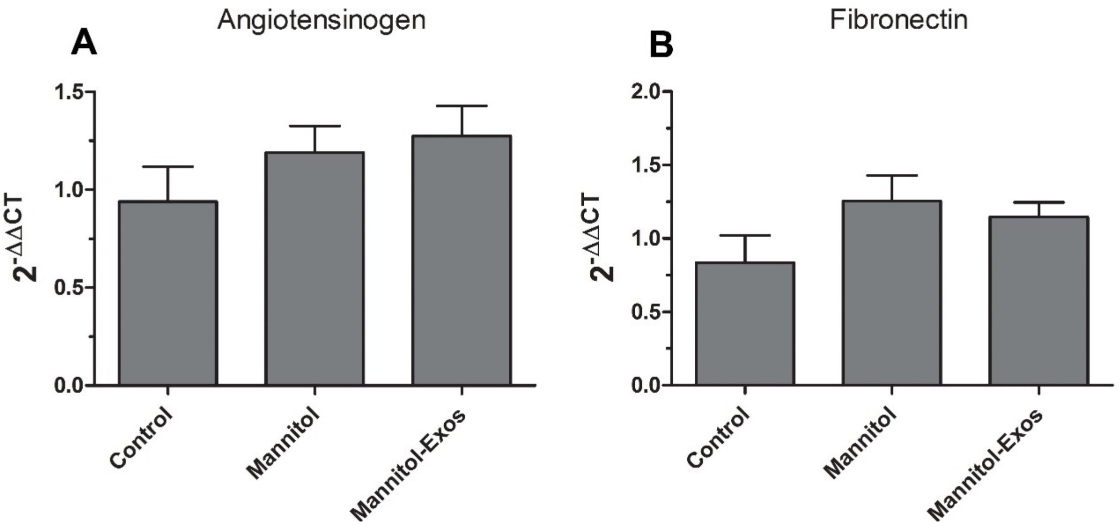

Supplemental Figure 5 - Real-time PCR for angiotensinogen and fibronectin in HMCs. Normal HMCs were incubated during 24 hr with mannitol (30mM) or with Exos derived from mannitol-stimulated HMCs (mannitol-Exos). After 24 hr, the gene expressions for angiotensinogen (AGT) (A) and fibronectin (B) were evaluated by real time PCR. The results are expressed as the means ± standard error of the mean (SEM).

Unprocessed Original Scans for all of the blots in figures 2, 4, 6 and 7

Figure 2E

CD63

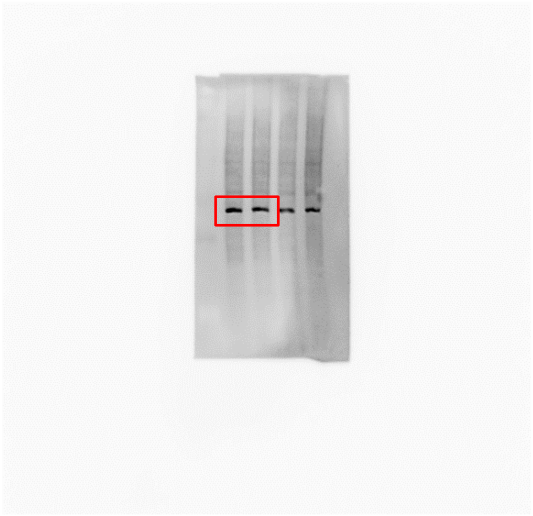

CD81

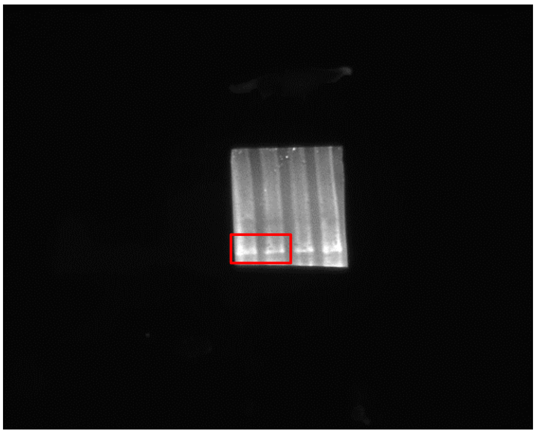

Calnexin

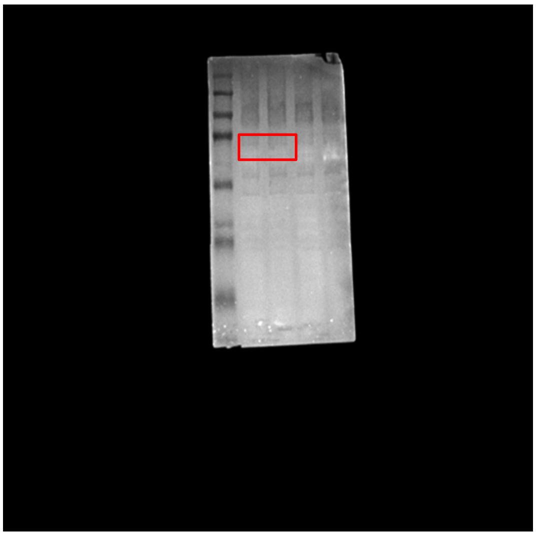

Figure 4 A

AGT

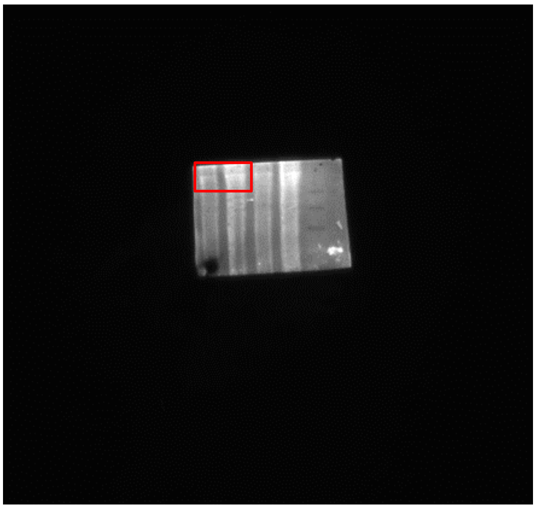

Renin

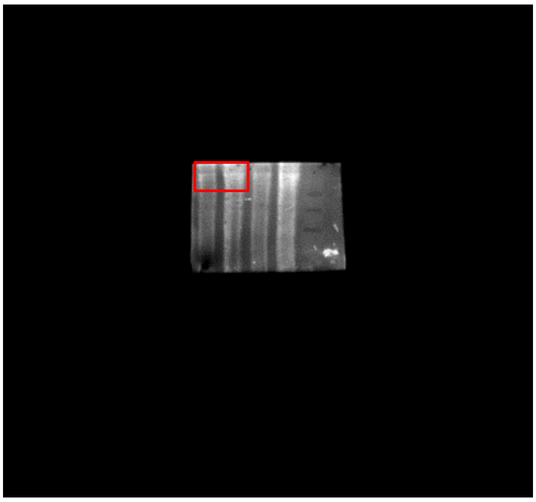

CD63

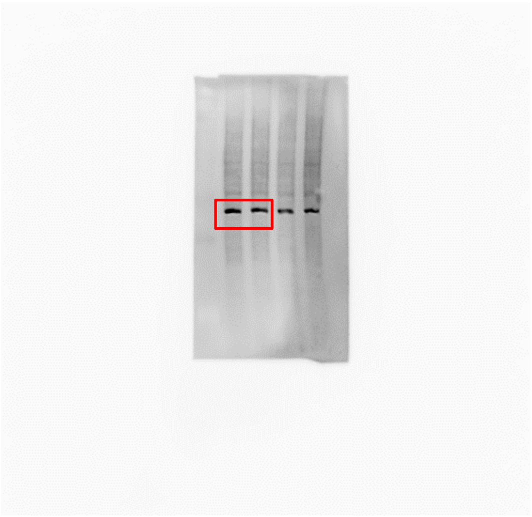

Figure 4 D

ACE

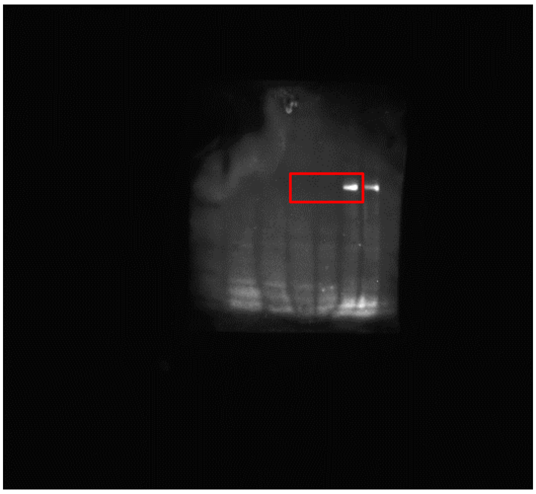

CD63

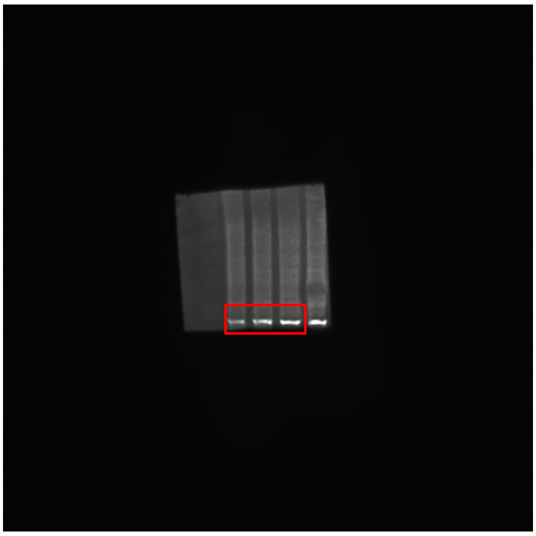

Figure 6E

AT$_1$

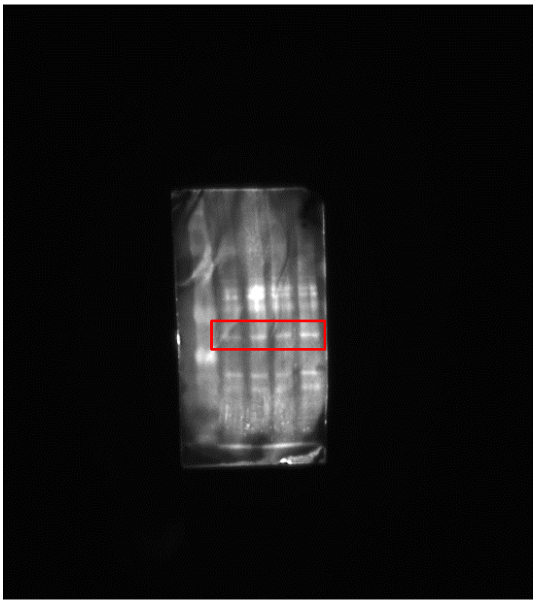

AT_2

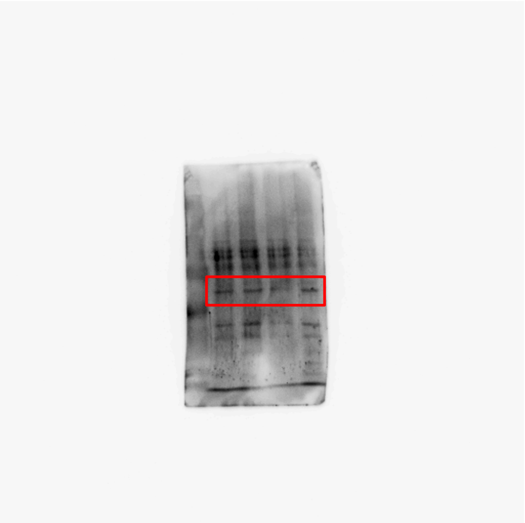

Histone

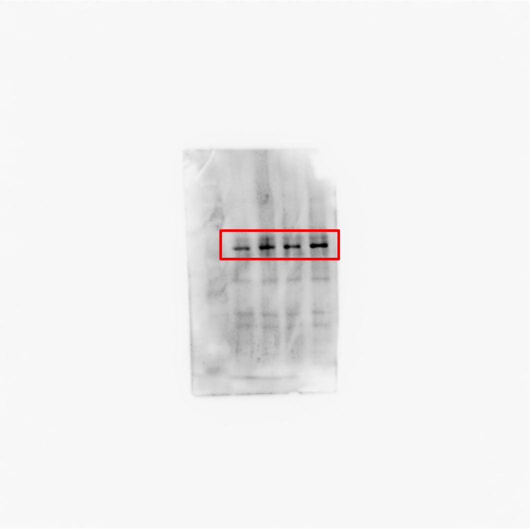

α - actin

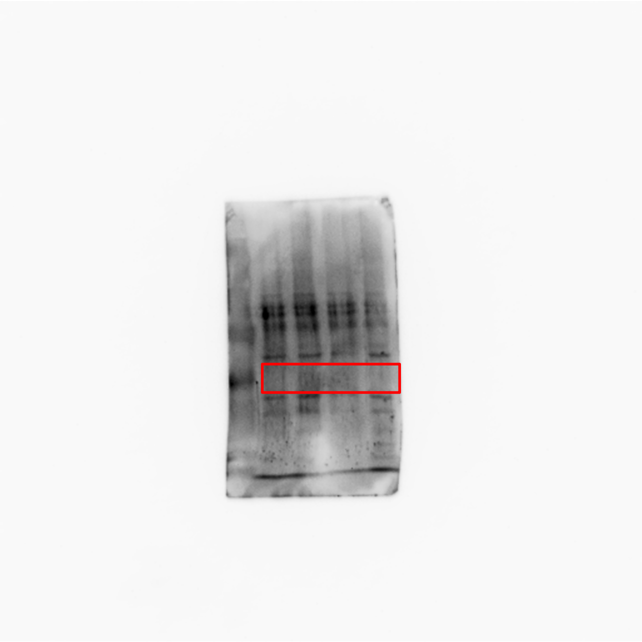

S100A10

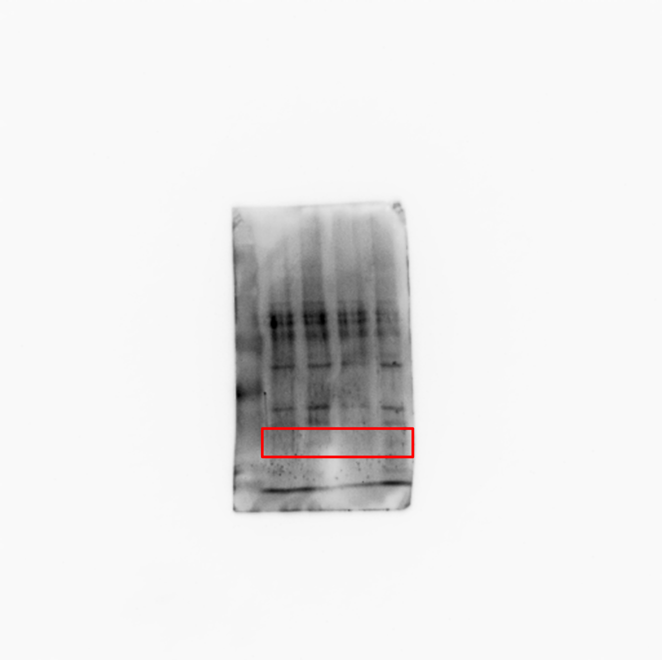

Figure 7G

AGT

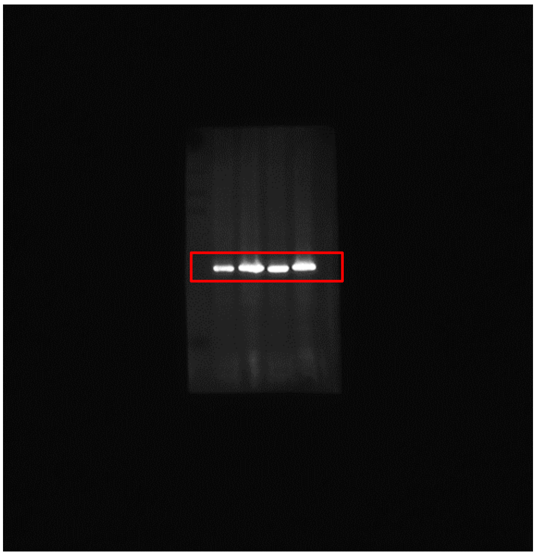

Renin

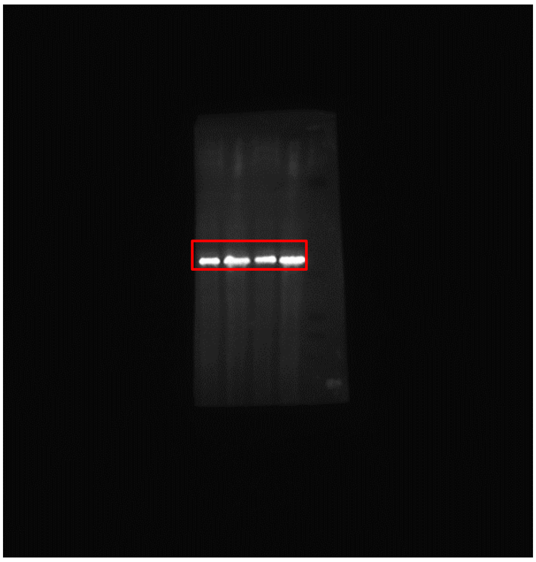

AT_1

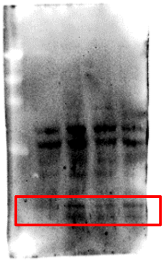

AT_2

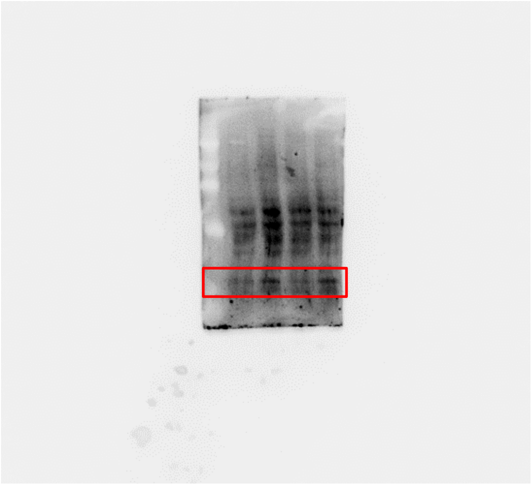

Beta actin

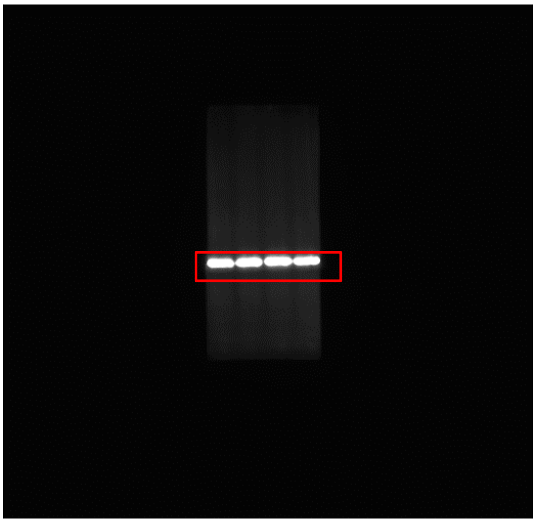

Supplement: Supplementary file 1 — Supplemental Figures [file 41598_2019_42746_MOESM1_ESM.pdf]
